# Resolution of P-Sterogenic 1-Phenylphosphin-2-en-4-one 1-Oxide into Two Enantiomers by (*R*,*R*)-TADDOL and Conformational Diversity of the Phosphinenone Ring and TADDOL in the Crystal State

**DOI:** 10.3390/molecules26226873

**Published:** 2021-11-15

**Authors:** Elżbieta Łastawiecka, Adam Włodarczyk, Anna E. Kozioł, Hanna Małuszyńska, K. Michał Pietrusiewicz

**Affiliations:** 1 Department of Organic Chemistry, Institute of Chemical Sciences, Faculty of Chemistry, Maria Curie-Sklodowska University, Gliniana 33, 20-614 Lublin, Poland; elzbieta.lastawiecka@mail.umcs.pl (E.Ł.); adam.wlodarczyk@mail.umcs.pl (A.W.); 2 Department of Crystallography, Institute of Chemical Sciences, Faculty of Chemistry, Maria Curie-Sklodowska University, Maria Curie-Sklodowska sq. 3, 20-031 Lublin, Poland; anna.koziol@mail.umcs.pl; 3 Faculty of Physics, Adam Mickiewicz University, Umultowska 85, 61-614 Poznań, Poland; hanmal@amu.edu.pl

**Keywords:** P-stereogenicity, phosphinanes, 1-phenylphosphin-2-en-4-one 1-oxide, six-membered carbon-phosphorus heterocycles, resolution, absolute configuration, TADDOL, optically active phosphines

## Abstract

The resolution of racemic 1-phenylphosphin-2-en-4-one 1-oxide (**2**), was achieved through the fractional crystallization of its diastereomeric complexes with (4*R*,5*R*)-(−)-2,2-dimethyl -α,α,α′,α′-tetraphenyl-dioxolan-4,5-dimethanol (*R,R*-TADDOL) followed by the liberation of the individual enantiomers of **2** by flash chromatography on silica gel columns. The resolution process furnished the two enantiomers of **2** of 99.1 and 99.9% e.e. at isolated yields of 62 and 59% (counted for the single enantiomer), respectively. The absolute configurations of the two enantiomers were established by means of X-ray crystallography of their diastereomerically pure complexes, i.e., (*R*)-**2**•*R*,*R*)-TADDOL and (*S*)*-***2**•(*R*,*R*)-TADDOL. The structural analysis revealed that in the (*R*)-**2**•(*R*,*R*)-TADDOL complex, the P-phenyl substituent occupied a pseudoequatorial position, whereas in (*S*)-**2**•(*R*,*R*)-TADDOL, it appeared in both the pseudoequatorial and the pseudoaxial positions in four symmetrically independent molecules. Concurrent conformational changes of the TADDOL molecules were best described by the observed changes of a pseudo-torsional CO...OC angle that could be considered as a possible measure of TADDOL conformation in its receptor–ligand complexes. The structural analysis of the (*R*,*R*)-TADDOL molecule revealed that efficiency of this compound for use as an effective resolving factor comes from its ability to flexibly fit its structure to both enantiomers of a ligand molecule, producing a rare case of resolution for both pure enantiomers with one chiral separating agent. The resolved (*R*)-**2** was used to assign the absolute configuration of a recently described (−)-1-phenylphosphin-2-en-4-one 1-sulfide by chemical correlation. In addition, an attempted stereoretentive reduction of (*R*)-**2** by PhSiH_3_ at 60 °C revealed an unexpectedly low barrier for P-inversion in 1-phenylphosphin-2-en-4-one.

## 1. Introduction

**Figure 1:**
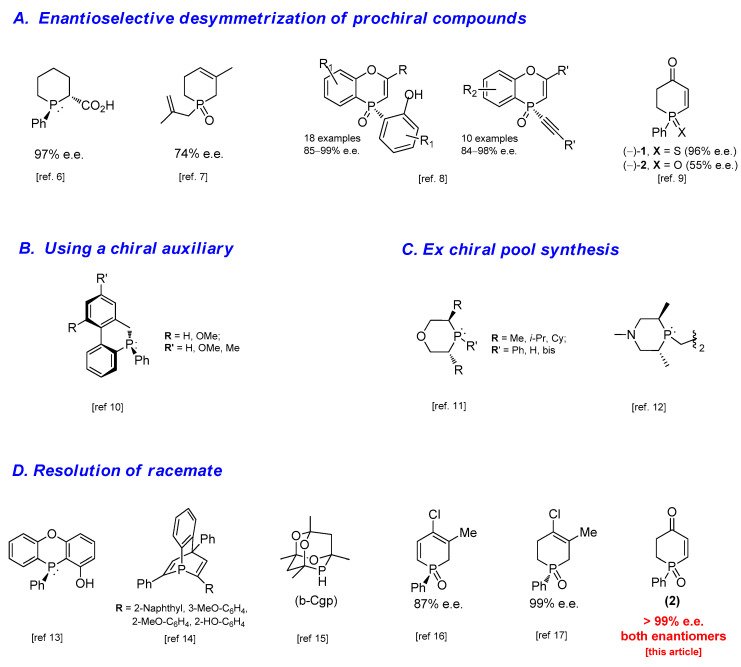
Reported optically active phosphinanes and type of reactions used in their synthesis.

The birth and evolution of asymmetric homogeneous catalysis is strongly coupled to the development of chiral phosphorus ligands. Among them, chiral phosphacyclic ligands of different ring extensions ranging from four to seven units have been identified as the most important ones [1,2,3,4,5]. One of the least represented groups of phosphorus heterocyclic ligands are chiral six-membered carbon-phosphorus heterocycles (phosphinanes). Despite intensive research efforts over the past two decades, nonracemic phosphinanes are still relatively scarce due, most probably, to the deficiency of convenient methods enabling the synthesis of functionalized phosphinane rings that are suitable for their further structural modifications [6,7,8,9,10,11,12,13,14,15,16,17]. A collection of all of the known optically active phosphinanes grouped by their preparation methods is presented in Figure 1.

As seen in Figure 1, one of the elegant ways in which to access nonracemic P-stereogenic phosphinanes is the use of asymmetric catalysis, which in this case, is predominantly based on the asymmetric desymmetrization of an extant phosphinane ring or on asymmetric cyclization of suitably functionalized prochiral substrates (Figure 1A) [6,7,8,9]. Other syntheses have relied on the use of a stoichiometric amount of a chiral auxiliary [10] or on chiral pool skeletal modifications (Figure 1B,C) [11,12]. Finally, the classical resolution of preformed racemic phosphinanes has also been successfully used to yield a series of optically active mono- and tricyclic phosphinanes (Figure 1D) [13,14,15,16,17].

Recently, we showed that optically active 1-phenylphosphin-2-en-4-one 1-sulfide (**1**) of 96% e.e. can be obtained via the asymmetric deprotonation of 1-phenylphosphinan-4-one 1-sulfide by a chiral base followed by the IBX•MPO-promoted oxidation of the intermediate silyl enol ether [9]. In a similar desymmetrization process, the corresponding phosphinanone oxide gave the desired 1-phenylphosphin-2-en-4-one 1-oxide (**2**) of only 55% e.e. [9]. As such, we have decided to attempt to access the phosphinanone oxide **2** with ahigh optical purity by means of classical resolution, which is readily scalable and, in a favorable case, can even deliver both enantiomers of **2** in a single resolution run.

In this paper, we aim to achieve this through the use of (*R,R*)-TADDOL as a resolving agent, which has been found to form crystalline diastereomeric complexes of >99% d.e. with both enantiomers of **2**. We shall also present and analyze the differences in interactions and conformational changes of the (*R,R*)-TADDOL and phosphinenone enantiomers because the propitious crystallization made the determination of the crystal structure of the both diastereomerically pure complexes possible.

## 2. Results and Discussion

### 2.1. Synthesis and Resolution of 1-Phenylphosphin-en-4-one 1-oxide (***2***)

**Scheme 1:**
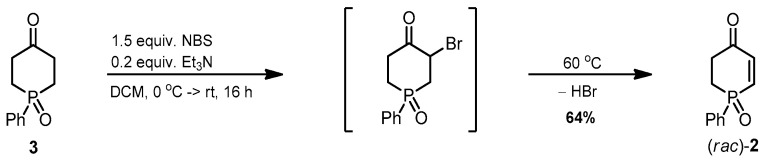
Synthesis of racemic 1-phenylphosphin-2-en-4-one 1-oxide (**2**) by the α-bromination/elimination route.

The synthesis of racemic **2** was conveniently accomplished by the α-bromination of 1-phenylphosphinan-4-one 1-oxide (**3**) followed by the thermal in situ dehydrobromination of the formed 3-bromo-1-phenylphosphinan-4-one 1-oxide yielding phosphinenone *rac*-**2** in good isolated yield (Scheme 1).

Having secured a convenient method for the synthesis of gram quantities of *rac*-**2,** we then attempted to elaborate an effective method for its resolution.

Due to our previous experience, we first tried L-menthyl bromoacetate and DBTA as resolving agents. These two reagents recently allowed us to successfully resolve 1-phenylphosphol-2-ene [18,19] and secondary *tert*-butyl-phenylphosphine oxide [20], respectively, providing both enantiomers of the corresponding phosphine oxide of very high enantiomeric purity in a single resolution process in each case. Unfortunately, the attempted use of these reagents for the resolution of phosphinenone **2** did not result in the formation of complexes suitable for crystallization.

**Figure 2:**
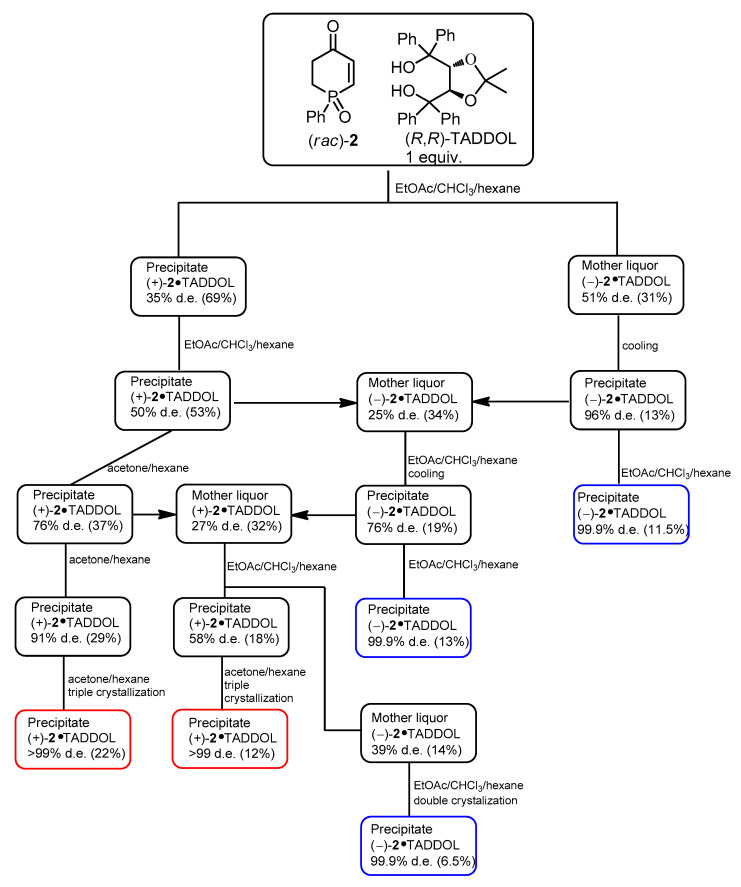
Gram-scale resolution of 1-phenylphosphin-en-4-one 1-oxide (**2**) by (*R*,*R*)-TADDOL.

Subsequent trials with TADDOL, another resolving agent which has already successfully been utilized for the separation of racemic phospholene and phosphinane oxides by Keglevich et al. [17,21,22,23], finally led to the development of an efficient resolution procedure for phosphinenone oxide **2**. The developed protocol for the resolution of phosphinenone oxide **2** by (*R*,*R*)-TADDOL is detailed in Figure 2.

To start the resolution process, a hot solution of the equimolar amounts of *rac*-**2** (9 g) and (*R*,*R*)-TADDOL (19 g) in a mixture of ethyl acetate and chloroform was treated with hexane to result in the precipitation of a crystalline complex of (+)-**2** • (*R*,*R*)-TADDOL (1:1) within 12 h.

The enantiomeric purity of the molecular complex (*+*)-**2**•(*R*,*R*)-TADDOL obtained in this way was 35% d.e. (69% yield). The additional recrystallization of this complex from the same mixture of solvents increased the d.e. to 50% (53%). Due to the fact that a further increase in the diastereomeric purity proved to be difficult to achieve, after a few trials, the solvent mixture was changed to acetone/hexane, which allowed the d.e. of (+)-**2**•(*R*,*R*)-TADDOL to increase this complex up to >99% (22%).

When the mother liquor of the first crystallization was cooled down and stored in a freezer (at −10 °C) overnight, the other diastereomer, i.e., (−)-2•(*R*,*R*)-TADDOL, of 96% d.e. was obtained in a 13% yield. Another recrystallization of this complex from a mixture of ethyl acetate/chloroform/hexane at room temperature afforded a (−)-**2**•(*R*,*R*)-TADDOL complex of 99.9% d.e. at an 11.5% yield.

In addition, the appropriate combination of mother liquors and continued fractional crystallizations from properly adjusted solvent mixtures, as indicated in the middle part of Figure 2, allowed us to increase the total yield of both the virtually diastereomerically pure complexes, i.e., (+)-**2**•(*R*,*R*)-TADDOL and (−)-**2**•(*R*,*R*)-TADDOL, to 34 and 31%, respectively.

It is also worthy of noting that the large difference in the retention times of TADDOL and phosphine oxide **2** allowed for very convenient CSP-HPLC monitoring of the d.e. (via e.e. of **2**) of the separated complexes that were present in each fraction during the entire resolution process.

**Figure 3:**
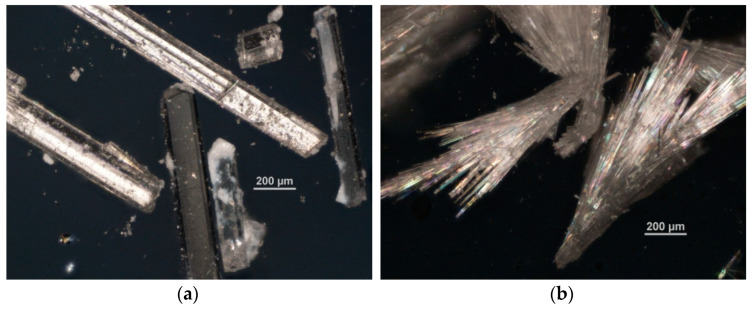
Images of the habit of crystals: (**a**) (−)-**2**•(*R*,*R*)-TADDOL and (**b**) (+)-**2**•(*R*,*R*)-TADDOL under polarized light microscopy.

Separated crystals of diasteromerically pure complexes of 1-phenylphosphin-en-4-one 1-oxide (**2**) with (*R*,*R*)-TADDOL were examined by means of polarized light microscopy (Figure 3).

Depending on the enantiomer bonded with TADDOL, the crystals take on a different morphology. The (−)-**2**•(*R,R*)-TADDOL crystals have a columnar tendency (Figure 3a), whereas needle-shaped (+)-**2**•(*R,R*)-TADDOL crystals are soft and form twins along the long edges (Figure 3b). Both of the crystals are colorless and transparent.

### 2.2. Structure of the Molecular Complexes (−)-2•(R,R)-TADDOL and (+)-2•(R,R)-TADDOL and Assignment of the Absolute Configuration of Enantiomers of ***2***

**Figure 4:**
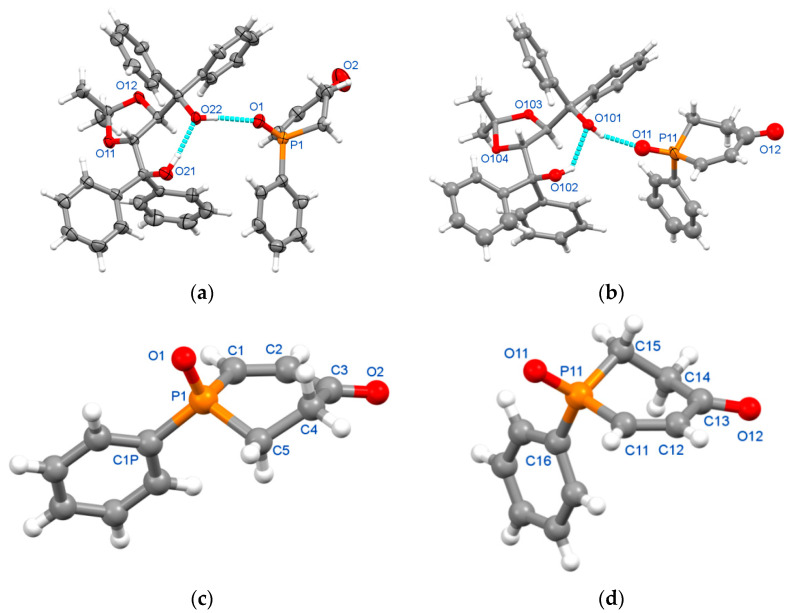
The 1:1 structure of the molecular complexes of (*R*,*R*)-TADDOL with ®-**2** (**a**) and (*S*)-**2** (**b**) with thermal ellipsoids set at 50% probability. Perspective view of enantiomeric molecules ®-**2** (**c**) and (*S*)-**2** (**d**). Dotted blue lines indicate the O-H...O hydrogen bonds.

**Figure 5:**
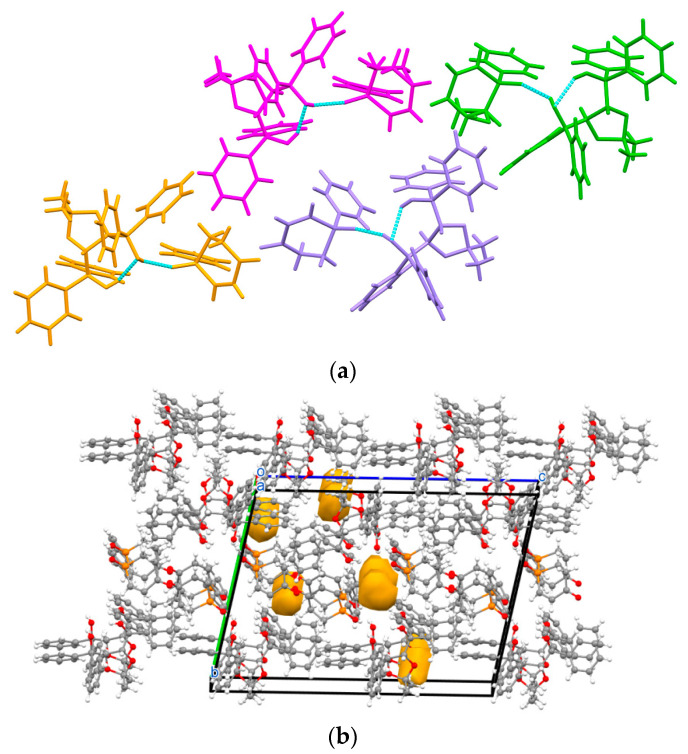
View of (*S*)-**2**•(*R,R*)-TADDOL: (**a**) the symmetrically independent part (Z’ = 4) of the crystal and (**b**) empty voids (1.3% of unit cell volume) in the crystal structure.

The crystals of diastereoisomerically pure complexes of enantiomers of **2** with (*R*,*R*)-TADDOL were subjected to a single-crystal X-ray analysis to determine their structure and absolute configuration. The complexes contained (*R*,*R*)-TADDOL and phosphine oxide **2** in a 1:1 ratio (Figure 4), which was in accordance with the NMR studies. The molecular complex (−)-**2**•(*R,R*)-TADDOL crystallizes in the orthorhombic crystal system in the chiral *P*2_1_2_1_2_1_ space group. The molecular complex (*+*)-**2**•(*R,R*)-TADDOL crystallizes in the triclinic chiral *P*1 space group with four symmetrically independent chemical units. Thus, the stoichiometry in this crystal is 4:4. When the crystal unit cells have a content such as this, it enables the better fulfillment of the need for the cells to be as densely packed into the space as possible, which is achieved by conformational changes in the molecules. However, the crystal quality is poor, and two of the four phosphinenone molecules are disordered. As a result, this enantiomer of **2** is “frozen” solid in several conformations of the phosphinenone ring, and voids are observed in the crystal (Figure 5).

The absolute configuration of the phosphine oxide in the (-)-**2**•(*R,R*)-TADDOL complex forming the thick good quality columnar crystals with (*R,R*)-TADDOL was determined as *R.* The molecular structure of the complex and of (*R*)-**2** are presented in Figure 4a,c. Accordingly, and as confirmed independently by an X-ray analysis of the second complex, the opposite enantiomer of **2** could be assigned to have the *S* configuration at the phosphorus atom (Figure 4b,d).

**Figure 6:**
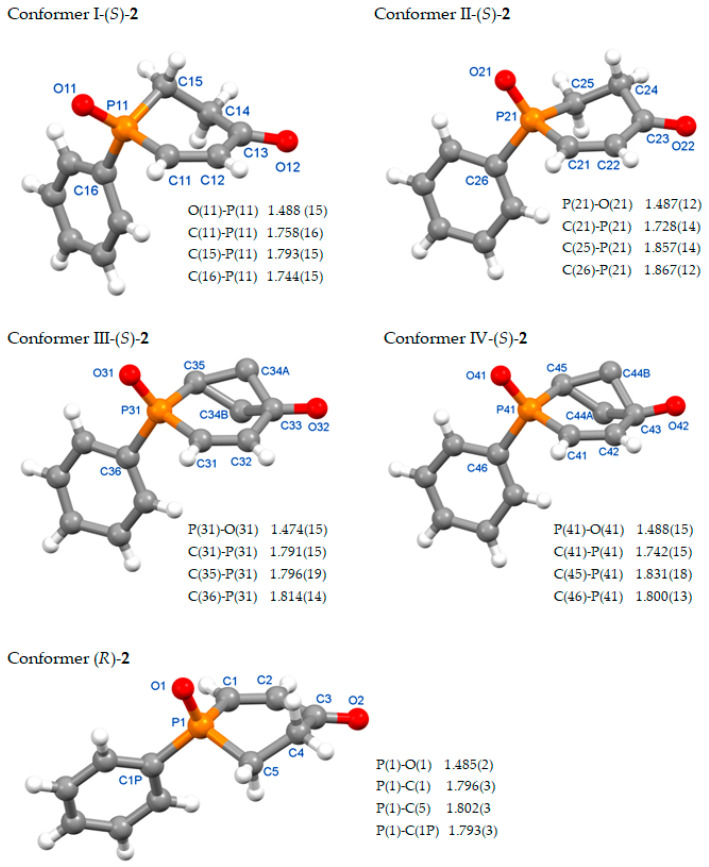
Perspective view of solid-state conformers observed for enantiomers *S* and *R* with bond lengths involving the P atom. The conformers III-(*S*)-**2** and IV-(*S*)-**2** are disordered.

In the (*S*)-**2**•(*R,R*)-TADDOL crystals, two of the enantiomer (*S*)-**2** positions are ordered (conformers I-(*S*)-**2** and II-(*S*)-**2**), while the other two are occupied by molecules, showing a statistical disorder between the two conformations (conformers III-(*S*)-**2** and IV-(*S*)-**2**), as depicted in Figure 6.

**Figure 7:**
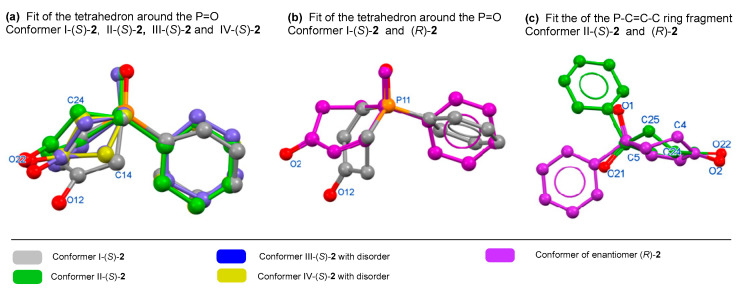
Molecular fitting of selected conformers. H-atoms are omitted for clarity.

The conformational variation of the six-membered phosphinenone ring takes place by changing the position of the C_sp3_-C_sp3_ atoms. In order to provide a complete description of the phosphinenone conformation, the specification of the endocyclic torsion angles within the rings was completed (Table 1). In the solid state, the (*S*)-**2** molecules adopt six conformations that are in either a sofa or a half-chair formation. A ring substituent (phenyl group) at the phosphinenone unit can be either pseudoaxial or pseudoequatorial in the given conformer. The key parameter defining the orientation of the phenyl group in space are the exocyclic torsion angles, which are highlighted in green in Table 1. Interestingly, in the conformer I-(*S*)-**2,** the phenyl group adopts an energetically unpreferred pseudoaxial position [24]. Figure 7a provides the molecular fitting for all conformers of the *S* enantiomer observed in the unit cell of its complex with (*R,R*)-TADDOL.

The geometric data for the phosphinenone ring in molecule (*R*)-**2** indicate that it adopts a half-chair conformation with the P-phenyl group in the pseudoequatorial position (Figure 4c). A major conformational difference in the orientation of the phenyl ring is seen for I-(*S*)-**2** and (*R*)-**2** (Figure 7b). The molecular fitting of conformers II-(*S*)-**2** to (*R*)-**2**, which both adopt the half-chair conformation, shows the opposite twisting of the sp^3^-hybridised carbon atoms of the phosphinenone ring (Figure 7c).

### 2.3. Geometry of TADDOL Molecules and Their Intermolecular and Intramolecular Contacts

Due to its chemical structure, TADDOL, which possesses hydroxyl groups in the vicinity of the voluminous hydrophobic parts, is able to bind the specific chemical groups of the ligand. This type of molecular receptor•ligand complex can be formed with molecules with hydrogen bond acceptors, i.e., C=O, C=S, CN, or P=O groups, for example. Expectedly, the strongest directional interactions stabilizing the molecular complexes in the studied crystal structures are intermolecular O-H...O hydrogen bonds that are formed between one of the hydroxyl groups of TADDOL and the oxygen atom of P=O function of **2**. In the TADDOL molecule, there is also an intramolecular hydrogen bond between the hydroxyl groups, which helps to fine-shape a cavity for incoming ligand molecules (Figure 4a,c, Table 2).

**Figure 8:**
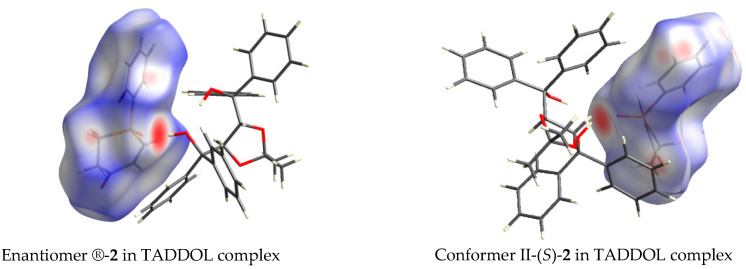
The Hirshfeld surfaces (d_norm_) for molecules (*R*)-**2** and II-(*S*)-**2.** The atoms with contacts shorter than the van der Waals radii are marked in red.

**Figure 9:**
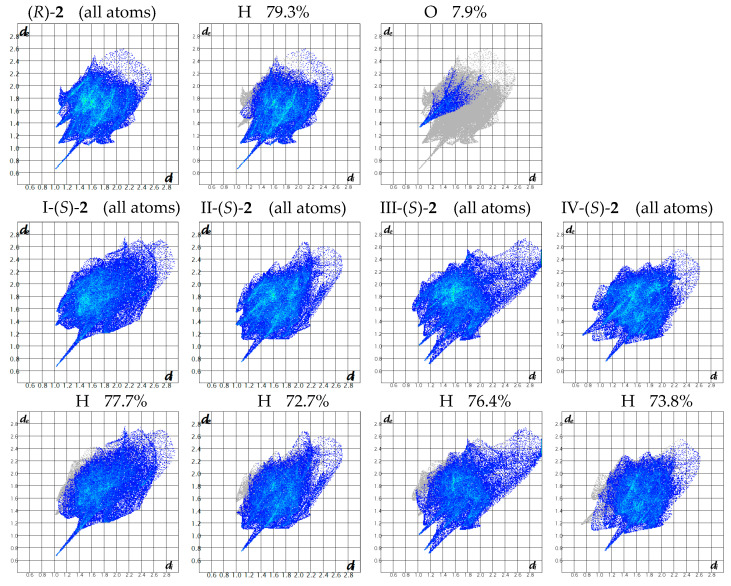
Fingerprints for intermolecular contacts involving enantiomeric (*R*)-**2** and (S)-**2** molecules.

Hirshfeld surfaces and their respective fingerprints [25] were calculated to illustrate the differences in the interactions involving the enantiomers (*R*)-**2** and (*S*)-**2** (Figure 8 and Figure 9). The dominant interactions are H...H contacts (72–79%), which is obvious when taking into account the chemical structure of the molecules. It should be emphasized that the range and directionality of the interactions of each S conformer are significantly different and are different from those of the *R* enantiomer.

**Figure 10:**
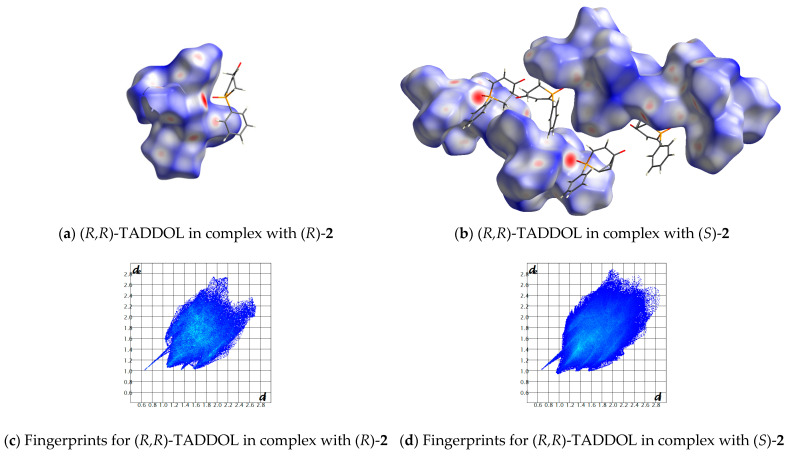
The Hirshfeld surfaces (d_norm_) for TADDOL molecules in complexes (**a**,**b**). The atoms with contacts shorter than the van der Waals radii are marked in red. Fingerprints for TADDOL interactions (**c**,**d**).

The Hirshfeld surfaces calculated for (*R,R*)-TADDOL (Figure 10) indicate that the hydroxyl group is a part of the O-H...O synthon.

**Figure 11:**
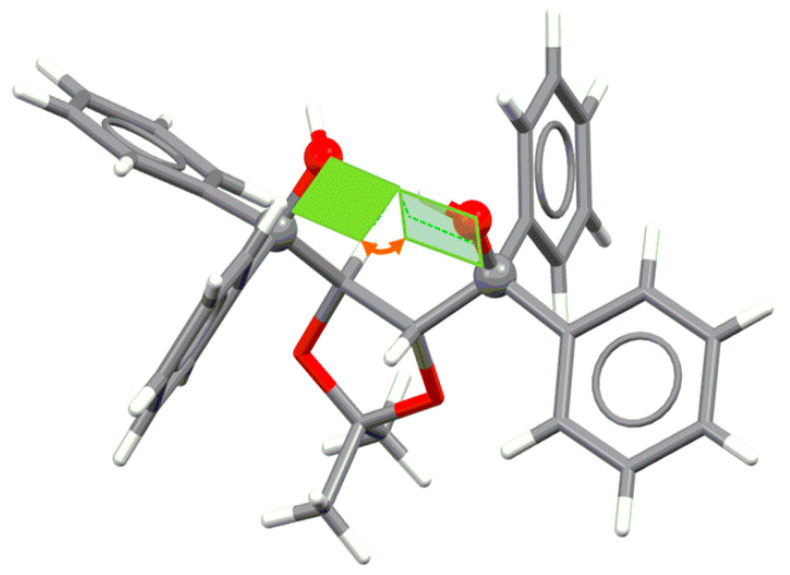
The definition of the C-O...O-C pseudo-torsion angle in TADDOL molecule.

The formation of a molecular complex of the receptor•ligand type, viz. the TADDOL•enantiomeric molecule, is possible due to a cavity formed inside the receptor molecule. The shape of this docking site can be changed by the rotation of the diphenyl- hydroxymethyl groups. To follow such conformational changes in the TADDOL molecule, in its complexes, we have chosen a pseudo-torsion angle C-O...O-C as a suitable geometric parameter (Figure 11).

As can be seen in Table 3, in the complexes described in this paper, the pseudo-torsion angles (Figure 11) were found to be in a rather narrow range of 14–21°, not indicating any really significant conformational changes in the TADDOL molecules. In order to compare the obtained results with the literature data, we did a structural data search in the Cambridge Structural Database (CSD ver. 5.42) for the occurrence of (*R*,*R*)-TADDOL complexes with various ligands. To our surprise, the search revealed only 46 determined crystal structures despite the fact that TADDOL is a rather frequently used resolving agent (see SI, for the results of the CSD search). Moreover, 11 crystals were solvated, and among the rest of the described structures, the crystals of a pair of diastereomeric complexes were only examined in two cases [26,27]. In addition, for one of them, the structure of a complex containing racemate was also determined.

An analysis of the collected structural data indicates that formation of a stable receptor•ligand system in the case of crystalline complexes of these diastereomeric pairs is accompanied by conformational changes in the TADDOL molecules. The values of the C-O...O-C pseudo-torsion angle in complexes formed with the *R* and with the *S* enantiomer can differ even by 38° within a pair (Table 3). As is also suggested by the sparse structural data that are available, another way to form stable receptor•ligand complexes is to increase the number of molecules in the asymmetric part of the cell (CUXKUC & CUXMIS) or to change the stoichiometry of the components (QERDEW & QERDIA).

Literature data for complexes with phosphine oxides only refer to two phospholene compounds (VIWFIR, HIBBUQ) [21,22]. In their fractional crystallization with TADDOL, crystals with only one enantiomer were formed, while the other remained in the mother liquor. The main difference between phospholene and phosphinenone is conformational rigidity. Apparently, in our study, a considerable conformational flexibility of the phosphinenone cyclic skeleton allowed the “mismatched” enantiomer to assume four conformations in order to achieve better fitting to the asymmetric environment at the docking point and to the crystal lattice. Altogether, it enabled the effective formation of a crystalline complex with this enantiomer, with only minor conformational changes in the TADDOL molecule.

### 2.4. Liberation of Enantiomers of 1-Phenyl-phosphin-2-en-4-one 1-oxide (***2***) from Their Diastereomerically Pure Complexes with (R,R)-TADDOL

**Figure 12:**
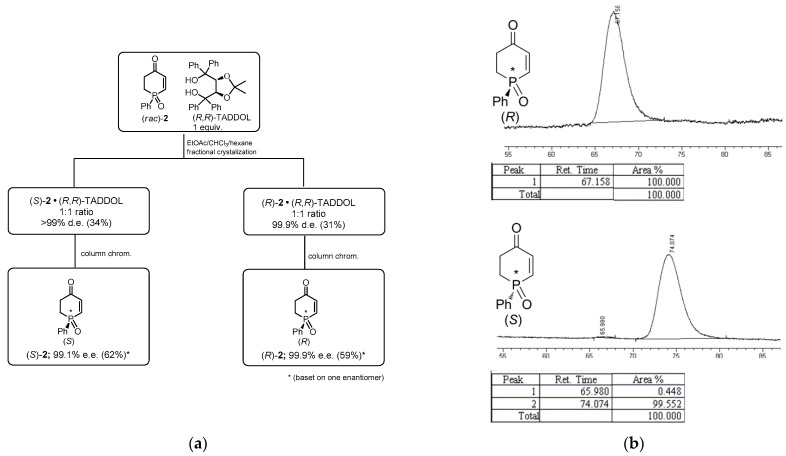
(**a**) Liberation of the enantiomers of phosphin-2-en-4-one **2** on silica gel columns; (**b**) copies of CSP-HPLC traces of (*R*)-**2** and *(S)-***2** (CHIRALCEL-OD-H).

Finally, the enantiomers of 1-phenyl-phosphinenone 1-oxide (**2**) were liberated from the diastereomerically pure complexes without any loss of optical purity at P by flash chromatography on silica gel (Figure 12). In this way, the enantiomers (*S*)-**2** (99.1% e.e.) and (*R*)*-***2** (99.9% e.e.) were obtained at overall yields of 62 and 59%, respectively, (based on one enantiomer), and the (*R,R*)-TADDOL was also recovered in a nearly quantitative yield.

### 2.5. Determination of the Absolute Configuration of 1-Phenylphosphin-2-en-4-one 1-Sulfide (***1***) by Chemical Correlation

**Scheme 2:**
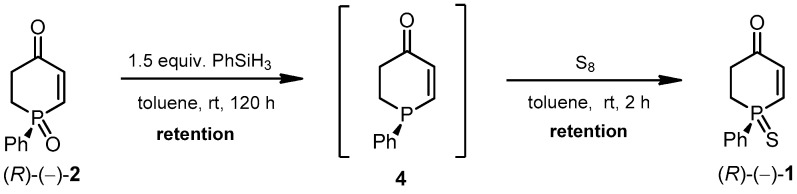
Chemical correlation of phosphinenone oxide ®-**2** with phosphinenone sulfide (−)-**1**.

As already mentioned above, we have previously obtained optically active 1-phenylphosphin-2-en-4-one 1-sulfide (−)-(**1**) of 96% enantiomeric purity by means of the catalytic desymmetrization of prochiral 1-phenylphosphinan-4-one 1-sulfide. However, due to the fact that it was an oil, its absolute configuration could not be readily determined. As we now have access to the resolved enantiomers of oxide **2** of the known configuration, we decided to carry out a chemical correlation of phosphinenone sulfide (−)-**1** with the resolved (−)-(*R*)-**2**. We chose to base the correlation on a stereoretentive reduction of phosphinenone oxide (*R*)-**2** by phenylsilane [28] followed by a similarly stereoretentive oxidation of the resulting phosphine **4** by elemental sulfur [29] to obtain phosphinenone sulfide (*R*)-**1** (Scheme 2).

**Figure 13:**
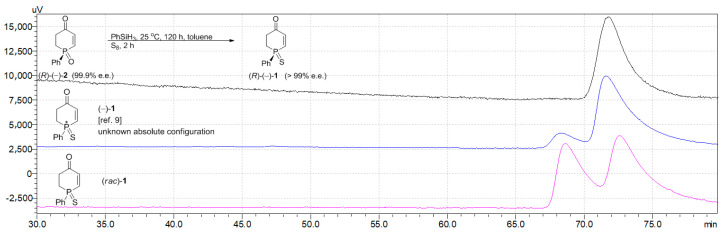
Assignment of the absolute configurations by chemical correlation. Copies of CSP-HPLC traces of—*rac-***1**;—(−)-**1**;—sulfide **1** from the correlation experiment.

To our surprise, carrying out the reduction of (*R*)-**1** under standard moderate conditions (heating at 60 °C for 48 h) resulted in obtaining a racemic phosphine **4**. We assumed that the observed racemization could be related to an unexpectedly low inversion barrier of the produced phosphine **4** [30]. Indeed, lowering the reduction temperature to 40 °C slowed down the inversion process as expected, but the partial erosion of the original 99.9% enantiopurity down to 41% e.e. was still observed. A full retention of the enantiomeric purity was finally achieved by running the reduction at room temperature, albeit at the cost of a very low yield (>99% e.e., 11% yield after 5 days). Then, to the crude reaction mixture containing enantiopure, phosphine **4** that had been obtained in this way was added to elemental sulfur to complete the correlation process (Scheme 2). A CSP-HPLC (CHIRALCEL OJ-H column) analysis of the produced phosphinenone sulfide (*R*)-**1** and the (−)-**1** previously obtained had revealed that the two phosphine sulfides were of the same configuration (Figure 13, top two traces). An additional run for racemic phosphinenone sulfide **1** (Figure 13, bottom trace) further corroborated this assignment. Therefore, the absolute configuration of the previously synthesized (−)-**1** could be unequivocally assigned as *R*.

## 3. Materials and Methods

### 3.1. General Information

Reagents were purchased from commercial suppliers and were used without further purification. All of the the reactions were performed in a flame dried, argon-filled glassware. Only dry, air-free solvents were used for the reactions: THF and toluene were dried over sodium/benzophenone ketyl; DCM was dried over P_4_O_10_. Solvents for chromatography and extraction were commercially available and used as received without further purification. Solvents for crystallization and NEt_3_ were distilled once before the use. The 1-phenyl-phosphinan-4-one 1-oxide [9] and TADDOL [31] were prepared according to procedures outlined in the literature.

The NMR spectra was recorded with a Bruker Ascend (500 MHz) spectrometer in CDCl_3_ as a solvent at room temperature unless otherwise noted. Chemical shifts (δ) are given in ppm relative to tetramethylsilane (^1^H), residual CHCl_3_ (^13^C) or external 85% H_3_PO_4_ (^31^P) as a reference. Coupling constants (*J*) are in Hz. The following abbreviations of signal patterns are as follows: s (singlet), d (doublet), t (triplet), q (quartet), m (multiplet), and br (broad). High-resolution mass spectrometry analyses were obtained on a Shimadzu LCMS IT-TOF spectrometer. Elementary analyses were performed on a PerkinElmer CHN 2400 analyzer. Melting points were determined on a Büchi Melting Point M-560 in a capillary tube and are uncorrected. Mass spectra were recorded with a GC-MS spectrometer working in electron ionization (EI) mode. Chiral HPLC analysis was performed on a Shimadzu HPLC using CHIRALCEL^®^ columns. Optical rotations were measured on a PerkinElmer 341LC spectrometer using a 1 mL cell with a 10 cm path length at ambient temperature and are reported as follows: ${\left[\alpha \right]}_{D}^{20}$ (c g/100 mL, solvent). Thin layer chromatography (TLC) was performed with precoated silica gel plates (Kieselgel 60, F254 on aluminum sheet, Merck) and visualized by potassium permanganate (KMnO_4_) stain or through exposure to iodine vapor. All separations and purifications by column chromatography were conducted using gel 60 (230–400 mesh), unless noted otherwise. X-ray crystallography: the single crystal diffraction data were collected with an Xcalibur Gemini diffractometer (Oxford Diffraction) using graphite monochromated CuKα radiation for the ®-**2**•TADDOL crystal and MoKα for the (*S*)-**2**•TADDOL complex. The CrysAlisPro program system [32] was used for data collection, cell refinement, and data reduction. The intensities were corrected for Lorentz and polarization effects, and a multi-scan absorption correction was applied. Crystal structure was solved by direct methods using the SHELXS-86 and SHELXT programs and refined by the full-matrix least squares method on *F*^2^ using the SHELXL-97 program [33,34,35]. The experimental details and final atomic parameters for the analyzed crystals were deposited into the Cambridge Crystallographic Data Centre as Supplementary Material.

### 3.2. Synthesis and Resolution of 1-Phenylphosphin-2-en-4-one 1-Oxide (***2***)

#### 3.2.1. Synthesis of (*rac*)-1-Phenylphosphin-2-en-4-one 1-Oxide (*rac*-**2**)

To a mixture of 1-phenylphosphinan-4-one 1-oxide (**3**) (1 g, 5 mmol) and Et_3_N (0.14 mL, 1 mmol) in DCM (100 mL) placed in a flame-dried Schlenk tube (200 mL) equipped with a magnetic stirring bar, 1.35 g (7.5 mmol) of NBS at 0 °C (ice/water bath) was added portionwise. Then, the resulting reaction mixture was allowed to warm to room temperature and was stirred for further 16 h at that temperature. After that time, the reaction mixture was warmed up to 60 °C (heating mantle) for 30 min to complete the quantitative elimination of HBr from the intermediate bromo ketone. The evaporation of the reaction mixture produced crude phosphinenone **2**. The crude product was purified on a silica gel column using DCM/THF = 10:1 as the eluent to produced 1-phenylphosphin-2-en-4-one 1-oxide (*rac-***2**) as colorless crystals at a64% yield (0.66 g, 3.2 mmol), m.p. = 106.5–107.3 °C, R_f_ = 0.33 (CHCl_3_/THF = 10:1). ^1^H NMR (500 MHz, CDCl_3_): *δ* 7.79–7.74 (m, 2H), 7.65–7.60 (m, 1H), 7.58–7.48 (m, 2H), 7.10–7.04 (m, 1H), 6.73 (dd, *J* = 13.6 and 23.0 Hz, 1H), 3.15 (s, 1H), 2.85–2.73 (m, 1H), 2.62–2.51 (m, 1H), 2.49–2.40 (m, 1H). ^13^C NMR (126 MHz, CDCl_3_): *δ* 196.1 (d, *J* = 14.5 Hz, C=O), 142.6 (C3), 138.2 (d, *J* = 83.6 Hz, C2), 132.9 (d, *J* = 2.7 Hz, C_para_), 130.6 (d, *J* = 10.9 Hz, C_ortho_), 129.9 (d, *J* = 105.4 Hz, C_ipso_), 129.2 (d, *J* = 12.7 Hz, C_meta_), 33.8 (d, *J* = 6.4 Hz, C5), 26.5 (d, *J* = 70.8 Hz, C6). ^31^P NMR (202 MHz, CDCl_3_): *δ* 16.9 ppm. GCMS (EI, 70 eV) *m*/*z* = 178.00 (33), 150.00 (19), 132.05 (10), 131.05 (100), 124.00 (24), 103.05 (14). Anal. Calcd for C_11_H_11_O_2_P: C, 64.08; H, 5.38. Found: C, 64.01; H, 5.24.

#### 3.2.2. Resolution of *rac*-**2** Using (*R,R*)-TADDOL

To 9 g (43.7 mmol) of 1-phenylphosphin-2-en-4-one 1-oxide (*rac*-**2**) and 19 g of *(R,R)-*TADDOL in 30 mL of hot CHCl_3_ and 20 mL of EtOAc, 75 mL of hexane were added. After the addition, coloreless crystals started to appear. The next 50 mL of hexane was added in two portions during following 4 h of the crystallization process. After 12 h, the crystals were separated by filtration to produce 20 g (69%) of complex (*S*)-**2**•*(R,R)-*TADDOL of 35% d.e. This complex was further purified by one recrystallization from a mixture of CHCl_3_/EtOAc/hexane (30/20/100 mL) to afford the complex (*S*)-**2**•*(R,R)-* TADDOL at a 53% yield and of 50% d.e. The next four recrystallizations of this complex from acetone/hexane (50/200 mL) allowed us to obtain the (*S*)-**2**•*(R,R)-* TADDOL complex at 99.9% d.e. and at a 22% yield. To obtain the opposite enantiomer of 1-phenyl-phosphinenone 1-oxide (**2**), the mother liquor of the first crystallization was cooled down and stored in a freezer (at −10 °C) for one night. During that time, the formation of (*R*)-**2**•*(R,R)-*TADDOL of 96% d.e. at a 13% yield was observed. The complex was further purified by one recrystallization from a mixture of CHCl_3_/EtOAc/hexane (10/10/70 mL) at room temperature to afford the complex (*R*)-**2**•*(R,R)-*TADDOL at 99.9% d.e. and at an 11.5% yield. The appropriate combination of mother liquors and the proper recrystallization of the selected solvent mixtures (see middle part of Figure 2) provided additional portions of diastereoisomerically pure (*S*)-**2**•*(R,R)-*TADDOL and (*R*)-**2**•*(R,R)-*TADDOL complexes, which increased the total yield for both the diastereoisomerically pure molecular complexes up to 34% and 31%, respectively. Column chromatography on silica gel (CHCl_3_/THF = 10/1) of the complexes furnished 2.65 g (12.9 mmol, 59% (based on one enantiomer)) of the enantiomerically pure (*R*)-1-phenyl-phosphinenone 1-oxide ((*R*)-**2**) and 2.78 g (13.5 mmol, 62% (based on one enantiomer)) of the >99% enantiomerically pure (*S*)-1-phenyl-phosphinenone 1-oxide ((*S*)-**2**). Importantly, the *(R,R)-*TADDOL that was quantitatively liberated in the same chromatographic isolation process was unaffected and could be recycled.

(*R*)-**2**•*(R,R)-*TADDOL complex; colorless crystals; m.p. = 159.6–160.7 °C; diastereomeric excess: >99% (${\left[\alpha \right]}_{D}^{20}$ = −128.8 (c 1.1, CHCl_3_)); CSP-HPLC conditions: CHIRALCEL OD-H, hexane/2-propanol = 90:10, 0.5 mL/min, retention time [for ®-**2**] = 66.6 min; Anal. Calcd for C_42_H_41_O_6_P: C, 74.98; H, 6.14; Found: C, 74.79; H, 6.17.

Crystal data: formula C_42_H_41_O_6_P, M_w_ = 672.72, crystal system orthorhombic, space group *P*2_1_2_1_2_1_. Unit cell dimensions: *a* = 9.3684(4) Å, *b* = 18.0344(6) Å, *c* = 20.8102(8) Å, V = 3516.0(2) Å^3^, Z = 4, D(calcd) = 1.271 g cm^−3^, μ(MoKα) = 1.082 mm^−1^, F(000) = 1424. Crystal size 0.02 × 0.03 × 0.25 mm^3^, λ = 1.5416 Å, θ = 3.243 to 68.255°, index ranges −11 ≤ *h* ≤ 10, −21 ≤ *k* ≤ 21, −25 ≤ *l* ≤ 25. Reflections collected/independent: 56559/6400 [R(int) = 0.0653]. Non-hydrogen atoms were refined with anisotropic displacement parameters. The positions of the O-bonded hydrogen atoms were found in a ΔF map and were refined isotropically. Carbon-bonded H-atoms were positioned geometrically and were allowed to ride on their parent atoms, with Uiso(H) = 1.5 Ueq for the methyl groups and 1.2 Ueq for the remaining C atoms. Parameters refined 452; Goodness-of-fit on F^2^ 1.062, final R indices [I > 2σ(I)] R1 = 0.0351, wR2 = 0.0752, R indices (all data) R1 = 0.0456, wR2 = 0.0808. Absolute structure parameter x = 0.007(9). Δρ max/min 0.17 and −0.23 e. Å^−3^. CCDC No. 2096677.

(*S*)-**2**•*(R,R)-*TADDOL complex; colorless crystals; m.p. = 158.1–158.6 °C; diastereomeric excess [of (*S*)-**2**]: >99%; (${\left[\alpha \right]}_{D}^{20}$ = +38.8 (c 1.0, CHCl_3_)); CSP-HPLC conditions: CHIRALCEL OD-H, hexane/2-propanol = 90:10, 0.5 mL/min, retention time [for (*S*)-**2**] = 74.3 min; Anal. Calcd for C_42_H_41_O_6_P: C, 74.98; H, 6.14; Found: C, 75.11; H, 6.20.

Crystal data: formula C_42_H_41_O_6_P, M_w_ = 672.72, crystal system triclinic, space group *P* 1. Unit cell dimensions: *a* = 8.538(2) Å, *b* = 18.162(3) Å, *c* = 23.912(4) Å, α = 98.12(1)°, β = 99.85(1)°, γ = 100.28(2)°, V = 3537.8(12) A^3^, Z = 4, D(calcd) = 1.263 g cm^−3^, absorption coefficient μ(MoKα) = 0.126 mm^−1^, F(000) = 1424. Crystal size 0.02 × 0.03 × 0.4 mm^3^, λ = 0.71073 Å, θ range for data collection 2.619 to 29.795°; index ranges −11 ≤ *h* ≤ 11, −24 ≤ *k* ≤ 13, −29 ≤ *l* ≤ 32. Reflections collected/independent [R(int) = 0.1153]/observed [I > 2σ(I)] 26584/19964/3973, completeness to θ = 25.242° 99.9%. The crystals diffracted poorly and the data-to-parameter ratio was very low at 4.9. Non-hydrogen atoms, with the exception of P atoms, were refined with isotropic displacement parameters. The model was refined with four disordered methylene C atoms, and 8 C-C bonds were constrained to 1.53 Å. Hydrogen atoms were positioned geometrically and retr allowed to ride on their parent atoms, with Uiso(H) = 1.5 Ueq for the hydroxyl and methyl groups and 1.2 Ueq for the remaining C atoms. Data/restraints/parameters 19964/11/812; Goodness-of-fit on F^2^ 0.661; Final R indices [I > 2σ(I)] R1 = 0.0827, wR2 = 0.1572; R indices (all data) R1 = 0.3070, wR2 = 0.2069; absolute structure parameter x = −0.1(2). Largest difference peak and hole 0.50 and −0.37 e. Å-3. CCDC No. 2096678.

(*R*)-1-phenyl-phosphorinenone 1-oxides (®-**2**); 99.9% e.e.; colorless crystals; m.p. = 128.5–129.1 °C; (${\left[\alpha \right]}_{D}^{20}$ = −284.1 (*c* 1.03, CHCl_3_)); R_f_ = 0.33 (CHCl_3_/THF = 10:1); Anal. Calcd for C_11_H_11_O_2_P: C, 64.08; H, 5.38; Found: C, 64.27; H, 5.43.

(*S*)-1-phenyl-phosphorinenone 1-oxides ((*S*)-**2**); 99.1% e.e.; colorless crystals; m.p. = 126.7–127.9 °C; (${\left[\alpha \right]}_{D}^{20}$ = +278.3 (*c* 1.1, CHCl_3_)); R_f_ = 0.33 (CHCl_3_/THF = 10:1); Anal. Calcd for C_11_H_11_O_2_P: C, 64.08; H, 5.38; Found: C, 64.14; H, 5.41.

### 3.3. Chemical Correlation of the Absolute Configurations of 1-Phenylphosphin-2-en-4-one 1-Sulfide (R)-***1***

An amount of 0.2 g (0.1 mmol) of (*R*)-1-phenyl-phosphin-2-en-4-one 1-oxide dissolved in 5 mL of dry toluene was placed in a 20 mL round-bottom flask. The solution was degassed and placed under an argon atmosphere. An amount of 0.185 mL (0.15 mmol) of PhSiH_3_ was added to the solution, and the reaction mixture was stirred at rt for 5 days. After this time, 0.03 g (0.1 mmol) of S_8_ was added to the solution, and the reaction mixture was stirred for 2 h. At the end of the reaction, the mixture was concentrated, and the crude product was passed through a silica gel column using hexane/THF (10:1) as the eluent to yield 0.022 g (11% overall yield after two steps) of 1-phenylphosphin-2-en-4-one 1-sulfide (*R*)-**1** as a colorless oil; (${\left[\alpha \right]}_{D}^{20}$ = −88.77 (*c* 1.1, CHCl_3_) for e.e. = 99%); R_f_ = 0.36 (hexane/THF = 6:1); CSP-HPLC conditions: CHIRALCEL OJ-H, hexane/2-propanol (95:5), 1 mL/min, retention time = 72 min. ^1^H NMR (500 MHz, CDCl_3_): *δ* 7.96–7.89 (m, 2H), 7.66–7.61 (m, 1H), 7.61–7.55 (m, 2H), 7.05–6.95 (m, 1H), 6.63 (dd, *J* = 12.5 and 34.5 Hz, 1H), 3.30 (tdd, *J* = 4.6, 12.2 and 16.5 Hz, 1H), 2.95–2.73 (m, 2H), 2.62–2.49 (m, 1H). ^13^C NMR (126 MHz, CDCl_3_): *δ* 195.6 (d, *J* = 13.6 Hz, C=O), 139.3 (d, *J* = 3.6 Hz, C3), 139.2 (d, *J* = 68.2 Hz, C2), 132.7 (d, *J* = 3.6 Hz, C_para_), 131.1 (d, *J* = 11.8 Hz, C_ortho_), 129.6 (d, *J* = 85.7 Hz, C_ipso_), 129.1 (d, *J* = 11.8 Hz, C_meta_), 34.0 (d, *J* = 7.3 Hz, C5), 31.3 (d, *J* = 55.4 Hz, C6). ^31^P NMR (202 MHz, CDCl_3_): *δ* 21.3 ppm. GCMS (EI, 70 eV) m/z = 223.05 (13), 222.05 (100), 190.10 (14), 189.10 (86), 171.10 165.05 (10), (12), 143.15 (12), 142.15 (96), 141.15 (18), 140.05 (50), 134.10 (22), 133.10 (50), 131.10 (20), 109.10 (11), 108.10 (28), 107.10 (69), 105.15 (24), 103.10 (18), 91.10 (10), 83.05 (12), 81.05 (11). Anal. Calcd for C_11_H_11_OPS: C, 59.45; H, 4.99. Found: C, 59.15; H, 4.78.

## 4. Conclusions

The developed resolution protocol provides ready and efficient access to gram quantities of both enantiomers of 1-phenylphosphin-2-en-4-one 1-oxide (**2**) through the separation of their molecular crystalline complexes with (*R*,*R*)-TADDOL. The recovery of the resolved enantiomers of **2** by flash column chromatography offers a convenient method for obtaining enantiomerically pure phosphine oxides (*R*)-**2** (59% yield, 99.9% e.e.) and (*S*)-**2** (62% yield, >99% e.e.) as well as for recovering (*R*,*R*)-TADDOL in a nearly quantitatively yield. The X-ray crystallography revealed that in the (*R*)-**2**•(*R*,*R*)-TADDOL complex, the P-phenyl substituent occupies a pseudoequatorial position, whereas in the (*S*)-**2**•(*R*,*R*)-TADDOL complex, it appears in both the pseudoequatorial and pseudoaxial positions in four symmetrically independent molecules. The resolved (*R*)-**2** was used to conduct a chemical correlation to assign the absolute configuration of a recently described (-)-1-phenylphosphin-2-en-4-one 1-sulfide (**1**) by chemical correlation. In addition, an attempted stereoretentive reduction of (*R*)-**2** by PhSiH_3_ at 60 °C revealed an unexpectedly low barrier for the P-inversion of 1-phenylphosphin-2-en-4-one.

The analysis of the structural data that have been published for the TADDOL molecule, which is frequently used as a receptor in racemate resolutions, so far showed that it is capable of adpating its conformation to the presence of opposite enantiomers of a ligand molecule. These changes enable the best steric adjustment of the receptor•ligand system, increasing the strength of hydrogen bonding to TADDOL in the solid. On the other hand, when the enantiomeric molecules are also conformationally flexible, they also adjust their shape to fit the TADDOL binding site. Experimental evidence was obtained from the crystallographic studies of the pair of enantiomeric TADDOL complexes analyzed in this work and two other analyses that were performed earlier.

## Figures and Tables

**Table 1:** Endocyclic torsion angles (°) for phosphinenone ring and exocyclic torsion angles describing orientation of substituents on P-atom in the enantiomeric molecules ®-**2** and (*S*)-**2**.

Conformer I-(*S*)-**2** (sofa)	angle	Conformer II*-*(*S*)-**2** (half-chair)	angle
P(11)-C(11)=C(12)-C(13)	−4(2)	P(21)-C(21)=C(22)-C(23)	−5(2)
C(11)=C(12)-C(13)-C(14)	−2(3)	C(21)=C(22)-C(23)-C(24)	21(2)
C(12)-C(13)-C(14)-C(15)	38(2)	C(22)-C(23)-C(24)-C(25)	−51(1)
C(13)-C(14)-C(15)-P(11)	−65(2)	C(23)-C(24)-C(25)-P(21)	61(2)
C(14)-C(15)-P(11)-C(11)	56(2)	C(24)-C(25)-P(21)-C(21)	−42(2)
C(15)-P(11)-C(11)=C(12)	−21(2)	C(25)-P(21)-C(21)=C(22)	15(2)
C(16)-P(11)-C(15)-C(14)	−59(2)	C(26)-P(21)-C(25)-C(24)	−153(3)
C(16)-P(11)-C(11)=C(12)	92(2)	C(26)-P(21)-C(21)=C(22)	124(2)
Conformer IIIB-(*S*)-**2** (sofa)	angle	Conformer IIIA-(*S*)-**2** (sofa)	angle
P(31)-C(31)=C(32)-C(33)	2(2)	P(31)-C(31)=C(32)-C(33)	2(2)
C(31)=C(32)-C(33)-C(34B)	−36(2)	C(31)=C(32)-C(33)-C(34A)	17(3)
C(32)-C(33)-C(34B)-C(35)	73(1)	C(32)-C(33)-C(34A)-C(35)	−41(3)
C(33)-C(34B)-C(35)-P(31)	−66(2)	C(33)-C(34A)-C(35)-P(31)	44(2)
C(34B)-C(35)-P(31)-C(31)	27(3)	C(34A)-C(35)-P(31)-C(31)	−26(3)
C(35)-P(31)-C(31)=C(32)	2(2)	C(35)-P(31)-C(31)=C(32)	2(2)
C(36)-P(31)-C(35)-C(34B)	−85(2)	C(36)-P(31)-C(35)-C(34A)	−138(2)
C(36)-P(31)-C(31)=C(32)	113(2)	C(36)-P(31)-C(31)=C(32)	113(2)
Conformer IVA(*S*)-**2** (half-chair)	angle	Conformer IVB-(*S*)-**2** (sofa)	angle
P(41)-C(41)=C(42)-C(43)	−11(2)	P(41)-C(41)=C(42)-C(43)	−11(2)
C(41)=C(42)-C(43)-C(44A)	21(2)	C(41)=C(42)-C(43)-C(44B)	42(3)
C(42)-C(43)-C(44A)-C(45)	59(2)	C(42)-C(43)-C(44B)-C(45)	−67(3)
C(43)-C(44A)-C(45)-P(41)	−61(2)	C(43)-C(44B)-C(45)-P(41)	59(3)
C(44A)-C(45)-P(41)-C(41)	32(3)	C(44B)-C(45)-P(41)-C(41)	−29(3)
C(45)-P(41)-C(41)=C(42)	5(2)	C(45)-P(41)-C(41)=C(42)	5(2)
C(46)-P(41)-C(45)-C(44A)	−80(2)	C(46)-P(41)-C(45)-C(44B)	−141(2)
C(46)-P(41)-C(41)=C(42)	114(2)	C(46)-P(41)-C(41)=C(42)	114(2)
*R* (half-chair)	angle		
P(1)-C(1)=C(2)-C(3)	−1.8(5)		
C(1)=C(2)-C(3)-C(4)	−8.7(5)		
C(2)-C(3)-C(4)-C(5)	44.5(4)		
C(3)-C(4)-C(5)-P(1)	−64.6(3)		
C(4)-C(5)-P(1)-C(1)	48.6(2)		
C(5)-P(1)-C(1)=C(2)	−18.1(3)		
C(1P)-P(1)-C(5)-C(4)	160.1(3)		
C(1P)-P(1)-C(1)=C(2)	−132.3(3)		

**Table 2:** Geometry of inter- and intramolecular O-H...O hydrogen bonds in molecular complexes of **2** with TADDOL acting as a hydrogen bond donor.

Acceptor Molecule	D-H...A	d(D-H) (Å)	d(H...A) (Å)	d(D...A) (Å)	<(DHA) (°)
I-(*S*)-**2**	O(101)-H(101)...O(11)	0.82	1.84	2.597(14)	153
(*)	O(102)-H(10G)...O(101)	0.82	2.04	2.687(13)	136
II-(*S*)-**2**	O(202)-H(20G)...O(21)	0.82	1.94	2.601(13)	137
(*)	O(201)-H(201)...O(202)	0.82	2	2.635(12)	134
III-(*S*)-**2**	O(301)-H(301)...O(31)	0.82	2	2.681(15)	140
(*)	O(302)-H(30G)...O(301)	0.82	2.05	2.670(13)	132
IV-(*S*)-**2**	O(401)-H(401)...O(41)	0.82	1.97	2.617(13)	135
(*)	O(402)-H(40G)...O(401)	0.82	2.03	2.696(12)	138
®-**2**	O(22)-H(11)...O(1)	0.84	1.83	2.659(5)	171
(*)	O(21)-H(21)...O(22)	0.9	1.75	2.645(4)	173

(*)—intramolecular hydrogen bond in TADDOL.

**Table 3:** The values of C-O...O-C angle in TADDOL observed in selected receptor•ligand complexes. (*)—crystal structures with Z’ = 2.

This Paper		Literature		Ref.
Conformer/Enantiomer	[°]	CSD REFCOD	[°]	
TADDOL∙ I-(*S*)-**2**	19	VIWFIR	15	[22]
TADDOL∙ II-(*S*)-**2**	14	HIBBUQ	24	[21]
TADDOL∙ III-(*S*)-**2**	14	CUXKUC (*S*)	44	[25]
TADDOL∙ IV-(*S*)-**2**	17	CUXMIS ®	30, 5	[25] (*)
TADDOL∙ ®-**2**	21	CUXLAJ (*rac*)	32, 3	[25] (*)
		QERDEW (*S*)	48	[26]
		QERDIA ® (2:1)	10, 12	[26]

## Data Availability

The data presented in this study are openly available.

## Supplementary Materials

Table S1: Data search in the Cambridge Structural Database (CSD ver. 5.42) for the occurrence of (*R*,*R*)-TADDOL complexes with various ligands [21,22,26,27,31,36,37,38,39,40,41,42,43,44,45,46,47,48,49,50,51,52,53,54,55,56].
