# Supplementary material for: Resolution of P-Sterogenic 1-Phenylphosphin-2-en-4-one 1-Oxide into Two Enantiomers by (R,R)-TADDOL and Conformational Diversity of the Phosphinenone Ring and TADDOL in the Crystal State
Source: Molecules. 2021 Nov 15;26(22):6873. doi: 10.3390/molecules26226873 (PMC8621017; doi:10.3390/molecules26226873)
Supplement: Supplementary file 1 [file molecules-26-06873-s001.zip › molecules-1461765-supplementary.pdf]

Supporting Information for article: Molecules, vol.: ; iss. ;

Resolution of P-sterogenic 1-Phenylphosphin-2-en-4-one 1-Oxide into two Enantiomers by (*R*,*R*)-TADDOL and Conformational Diversity of the Phosphinenone Ring and TADDOL in the Crystal State

Elżbieta Łastawiecka [1], Adam Włodarczyk [1], Anna E. Kozioł [2], Hanna Małuszyńska [3], and K. Michał Pietrusiewicz [1,*]

1 Department of Organic Chemistry, Institute of Chemical Sciences, Faculty of Chemistry, Maria Curie-Skłodowska University, Gliniana 33 str., 20-614 Lublin, Poland; lastawiecka@mail.umcs.pl; adam.wlodarczyk@mail.umcs.pl
2 Department of Crystallography, Faculty of Chemistry, Maria Curie-Skłodowska University Maria Curie-Skłodowska sq. 3, 20-031 Lublin, Poland; anna.koziol@mail.umcs.pl
3 Faculty of Physics, Adam Mickiewicz University, Umultowska 85, 61-614 Poznań, Poland; hanmal@amu.edu.pl
* Correspondence: kazimierz.pietrusiewicz@poczta.umcs.lublin.pl

Table S1. A search of the CSD database for (*R,R*)-TADDOL crystalline molecular complexes of receptor • ligand type.

No.	Ref. Code	Ligand	Ligand chirality	"receptor":"ligand" ratio	Solvate	Ref.
1.	CUXLAJ03	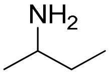	*rac*	1:1		[26]
2.	CUXKUC	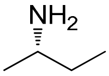	***S***	1:1		[26]
3.	CUXMIS	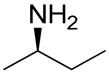	***R***	1:1		[26]
4.	LATCOY	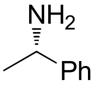	*S*	1:1		[36]
5.	DIFTIW	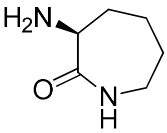	*S*	1:1		[37]
6.	RAHWUV	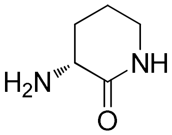	*R*	1:1		[38]
7.	HEZZUI	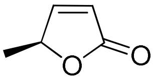	*S*	1:1		[39]
8.	HIBBAW	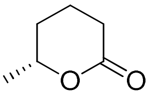	*R*	1:1		[39]
9.	WIRXEA	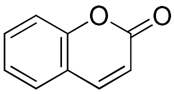	-	1:1		[40]
10.	WIRXIE	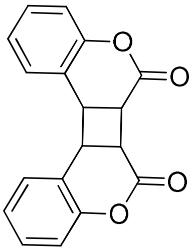	-	1:1		[40]
11.	KODWEF	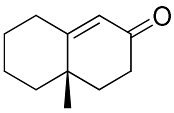	*R*	1:1		[41]

12.	KODWIJ	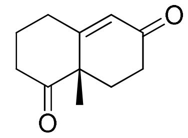	*R*	1:1		[41]
13.	KOMSAG	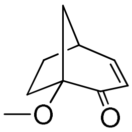	*R*	1:1		[42]
14.	QERDEW	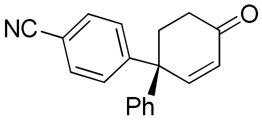	***S***	1:1		[27]
15.	QERDIA	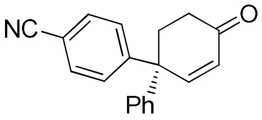	***R***	2:1		[27]
16.	RAZSUG	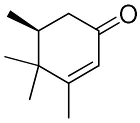	*S*	1:1		[43]
17.	FEMMAM	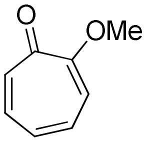	-	1:1		[44]
18.	HADKAZ	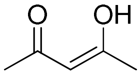	-	1:1		[45]
19.	NEMBOX	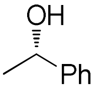	*S*	1:1		[46]
20.	MAWZUG	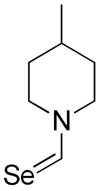	-	1:1		[47]
21.	MAXBAP	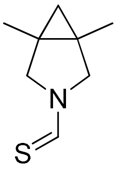	-	1:1	benzene	[47]
22.	MAXBET	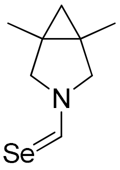	-	1:1	benzene	[47]

23.	MAXBOD	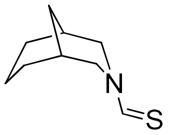	-	1:1		[47]
24.	MAXBUJ	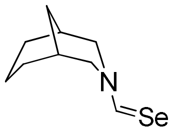	-	1:1		[47]
25.	UDIFUI	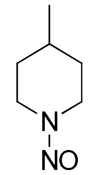	-	1:1		[48]
26.	UDIGET	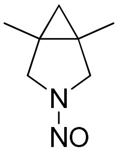	-	1:1		[48]
27.	UDIGIX	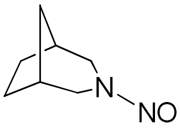	-	1:1		[48]
28.	VIMLOU	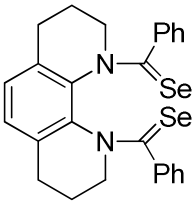	-	1:1		[49]
29.	VIMMEL	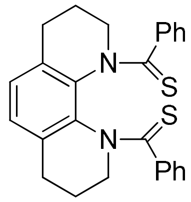	-	1:1		[49]
30.	VIMMIP	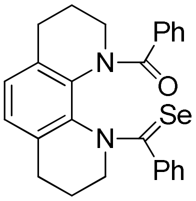	-	1:1		[49]
31.	NAHGIM	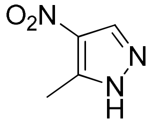	-	1:1	toluene	[50]

32.	TUTSOR	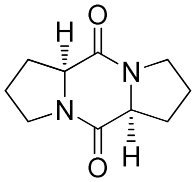	*S,S*	1:1		[51]
33.	HIBBUQ	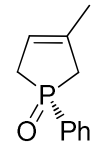	*S*	1:1	acetone	[21]
34.	VIWFIR	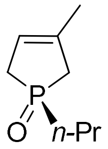	*R*	1:1		[22]
35.	NUYBOY01	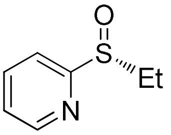	*R*	1:1		[52]
36.	RIBCIO	MeOH	-	2:1		[53]
37.	RIBCOU	EtOH	-	2:1		[53]
38.	SEWWIA	$EtNH_2$	-	1:1		[54]
39.	SEWWOG	Et_2NH	-	1:1		[54]
40.	SEWWUM	Et_3N	-	1:1		[54]
41.	TAMKEA	Pyridine	-	1:1		[55]
42.	TAMKIE	2-Methylpyridine	-	1:1		[55]
43.	TAMKOK	3-Methylpyridine	-	1:1		[55]
44.	TAMKUQ	4-Methylpyridine	-	1:1		[55]
45.	KOGJAR	CCl_4	-	1:4		[56]
46.	FAMJIN	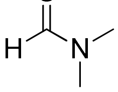	-	1:1		[31]

References for the article "Resolution of P-sterogenic 1-Phenylphosphin-2-en-4-one 1-Oxide into two Enantiomers by (*R*,*R*)-TADDOL and Conformational Diversity of the Phosphinenone Ring and TADDOL in the Crystal State"

1. Grabulosa, A. *P-Stereogenic Ligands in Enantioselective Catalysis*, 1st ed.; RSC Publishing: Cambridge, UK, 2011; pp. 1–501.
2. Kamer, P.C.J.; van Leeuwen, P.W.N.M. *Phosphorous(III) Ligands in Homogeneous Catalysis: Design and Synthesis*; John Wiley & Sons, Ltd.: West Sussex, UK, 2012.
3. Tang, W.; Zhang, X. New Chiral Phosphorus Ligands for Enantioselective Hydrogenation. *Chem. Rev*. **2003**, *103*, 3029−3069.
4. de Vries, J.G.; Elsevier, C.J. *Handbook of Homogeneous Hydrogenation;* Wiley-VCH: Weinheim, Germany, 2006; Volume 1−3.
5. Ni, H.; Chan, W.-L.; Lu, Y. Phosphine-Catalyzed Asymmetric Organic Reactions. *Chem. Rev*. **2018**, *118*, 9344−9411.
6. Kobayashi, S.; Shiraishi, N.; Lam, W.W.-L.; Manabe, K. Asymmetric synthesis of proline and pipecolic acid phosphorous analogues using enantioselective deprotonation−carboxylation reactions. *Tetrahedron Lett*. **2001**, *42*, 7303–7306.
7. Harvey, J.S.; Malcolmson, S.J.; Dunne, K.S.; Meek, S.J.; Thompson, A.L.; Schrock, R.R.; Hoveyda, A.H.; Gouverneur, V. Enantioselective Synthesis of P-Stereogenic Phosphinates and Phosphine Oxides by Molybdenum-Catalyzed Asymmetric Ring-Closing Metathesis. *Angew. Chem. Int. Ed*. **2009**, *48*, 762–766.
8. Zheng, Y.; Guo, L.; Zi, W. Enantioselective and Regioselective Hydroetherification of Alkynes by Gold-Catalyzed Desymmetrization of Prochiral Phenols with P-Stereogenic Centers. *Org. Lett*. **2018**, *20*, 7039–7043.
9. Łastawiecka, E.; Frynas, S.; Pietrusiewicz, K.M. Desymmetrization Approach to the Synthesis of Optically Active P-Stereogenic Phosphin-2-en-4-ones. *J. Org. Chem* **2021**, *86*, 6195–6206.
10. Mohar, B.; Čusak, A.; Modec, B.; Stephan, M. P-Stereogenic Phospholanes or Phosphorinanes from o-Biarylylphosphines: Two Bridges Not Too Far. *J. Org. Chem*. **2013**, *78*, 4665–4673.
11. Ostermeier, M.; Prieß, J.; Helmchen, G. Mono-and Bidentate Phosphinanes-New Chiral Ligands and Their Application in Catalytic Asymmetric Hydrogenations. *Angew. Chem. Int. Ed.* **2002**, *41*, 612–614.
12. Yan, Y.; Zhang, X. Six-membered bis(azaphosphorinane), readily available ligand for highly enantioselective asymmetric hydrogenations. *Tetrahedron Lett.* **2006**, *47*, 1567–1569.
13. Doro, F.; Lutz, M.; Reek, J.N.H.; Spek, A.L.; van Leeuwen, P.W.N.M. P-Chirogenic Benzo-Fused Phenoxaphosphane: Synthesis, Resolution and Study of the Stereochemical Properties of the Corresponding Palladium Complexes. *Eur. J. Inorg. Chem*. **2008**, *8*, 1309–1317.
14. Breit, B.; Fuchs, E. Chiral phosphabarrelene ligands: Synthesis and evaluation in rhodium-catalyzed asymmetric hydrogenation. *Synthesis* **2006**, *13*, 2121–2128.
15. Hopewell, J.; Jankowski, P.; McMullin, C.L.; Orpen, A.G.; Pringle, P.G. Subtleties in asymmetric catalyst structure: The resolution of a 6-phospha-2,4,8-trioxaadamantane and its applications in asymmetric hydrogenation catalysis. *Chem. Commun*. **2010**, *46*, 100–102.
16. Ujj, V.; Kerenyi, A.; Laki, A.; Fogassy, E.; Keglevich, G. Optically Active 6-Membered P-Heterocycles: 1-Phenyl-1,2- Dihydrophosphinine Oxide and 1-Phenyl-3-Diphenylphosphinoyl1,2,3,6-Tetrahydrophosphinine Oxide. *Lett. Org. Chem*. **2010**, *7*, 110−113.
17. Bagi, P.; Laki, A.; Keglevich, G. Preparation of Optically Active Six-Membered P-Heterocycles: A 3-Phosphabicyclo[3.1.0] hexane 3-oxide, a 1,2-Dihydrophosphinine 1-oxide, and a 1,2,3,6-Tetrahydrophosphinine 1-oxide. *Heteroat. Chem*. **2013**, *24*, 179–186.
18. Pietrusiewicz, K.M.; Koprowski, M.; Drzazga, Z.; Parcheta, R.; Łastawiecka, E.; Demchuk, O.M.; Justyniak, I. Efficient Oxidative Resolution of 1-Phenylphosphol-2-Ene and Diels–Alder Synthesis of Enantiopure Bicyclic and Tricyclic P-Stereogenic C-P Heterocycles. *Symmetry* **2020**, *12*, 346.
19. Pietrusiewicz, K.M.; Zablocka, M. Preparation of Scalemic P-Chiral Phosphines and Their Derivatives. *Chem. Rev.* **1994**, *94*, 1375–1411.
20. Holt, J.; Maj, A.M.; Schudde, E.P.; Pietrusiewicz, K.M.; Sieron, L.; Wieczorek, W.; Jerphagnon, T.; Arends, I.W.C.E.; Hanefeld, U.; Minnaard, A.J. On the Resolution of Secondary Phosphine Oxides via Diastereomeric Complex Formation: The Case of *tert*-Butylphenylphosphine Oxide. *Synthesis* **2009**, *12*, 2061–2065.
21. Novák, T.; Schindler, J.; Ujj, V.; Czugler, M.; Fogassy, E.; Keglevich, G. Resolution of 3-methyl-3-phospholene 1-oxides by molecular complex formation with TADDOL derivatives. *Tetrahedron: Asymmetry* **2006**, *17*, 2599–2602.
22. Novák, T.; Ujj, V.; Schindler, J.; Czugler, M.; Kubinyi, M.; Mayer, Z.A.; Fogassy, E.; Keglevich, G. Resolution of 1-substituted-3-methyl-3-phospholene 1-oxides by molecular complex formation with TADDOL derivatives. *Tetrahedron: Asymmetry* **2007**, *18*, 2965–2972.

23. Bagi, P.; Fekete, A.; Kállay, M.; Hessz, D.; Kubinyi, M.; Holczbauer, T.; Czugler, M.; Fogassy, E.; Keglevich, G. A Case Study on the Resolution of the 1-i-Butyl-3-methyl-3-phospholene 1-Oxide via Diastereomeric Complex Formation Using TADDOL Derivatives and via Diastereomeric Coordination Complexes Formed from the Calcium Salts of O,O′-Diaroyl-(2*R*,3*R*)-Tartaric Acids. *Heteroat. Chem.* **2014**, *26*, 79–90.
24. Von, L.D.; Quin, J. For earlier observations of axially positioned P-phenyl groups in phosphinane rings. In *The Heterocyclic Chemistry of Phosphorus: Systems Based on the Phosphorus-Carbon Bond*; John Wiley & Sons: New York, NY, USA, 1981; Chapter 8, pp. 368–370.
25. Spackman, M.A.; Jayatilaka, D. Hirshfeld surface analysis. *CrystEngComm* **2009**, *11*, 1081–1084.
26. Bathori, N.B.; Nassimbeni, L.R. Selectivity and Enantiomeric Resolution in Inclusion Chemistry: A Systematic Study of Chiral Discrimination through Crystallization. *Cryst. Growth Des.* **2010**, *10*, 1782–1787.
27. Zimmerman, H.E.; Alabugin, I.V.; Smolenskaya, V.N. Experimental and Theoretical Host–Guest Photochemistry; Control of Reactivity with Host Variation and Theoretical Treatment with a Stress Shaped Reaction Cavity; Mechanistic and Exploratory Organic Photochemistry. *Tetrahedron* **2000**, *56*, 6821–6831.
28. Marsi, K.L. Phenylsilane Reduction of Phosphine Oxides with Complete Stereospecificity. *J. Org. Chem.* **1974**, *39*, 265—267.
29. Horner, L.; Winkler, H. Phosphororganische Verbindungen XXXIX Konfigurationsbeziehungen zwischen optisch aktiven, Phosphororganischen Verbindungen. *Tetrahedron Lett.* **1964**, *5*, 175–179.
30. Holz, J.; Jiao, H.; Gandelman, M.; Börner, A. About the Inversion Barriers of P-Chirogenic Triaryl-Substituted Phosphanes. *Eur. J. Org. Chem.* **2018**, *23*, 2984–2994.
31. Du, H.; Zhao; D.; Ding, K. Enantioselective Catalysis of the Hetero-Diels-Alder Reaction between Brassard's Diene and Aldehydes by Hydrogen-Bonding Activation: A One-Step Synthesis of (S)-(+)-Dihydrokawain. *Chem. Eur. J.* **2004**, *10*, 5964–5970.
32. *CrysAlisPro, Version 1.171.36.20 (release 27–06–2012)*; Agilent Technologies Poland: Warsaw, Poland, 2012.
33. Sheldrick, G.M. SHELXS-86-A program for automatic solution of crystal structures. *Acta. Cryst*. **1990**, *A46*, 467–473.
34. Sheldrick, G.M. SHELXT-Integrated space-group and crystal-structure determination. *Acta Cryst*. **2015**, *A71*, 3–8.
35. Sheldrick, G.M. Crystal structure refinement with SHELXL. *Acta Cryst*. **2015**, *C71*, 3–8.
36. Toda, F.; Tanaka, K.; Ootani, M.; Hayashi, A.; Miyahara, I.; Hirotsu, K. Structure study of host–guest molecular association in solution and in the solid state. *J. Chem. Soc. Chem. Commun.* **1993**, *18*, 1413–1415.
37. Urbańczyk-Lipkowska, Z.; Fukuda, N.; Tanaka, K. Resolution of α-aminolactams by inclusion complexation with chiral host compounds. *Tetrahedron Asymmetry* **2007**, *18*, 1254–1256.
38. Lipkowska Z. CCDC 643248: Experimental Crystal Structure Determination. In *CSD Communication*; 2017. Available online: https://search.datacite.org/works/10.5517/ccdc.csd.ccplbyj (accessed on 20 October 2021).
39. Tanaka, K.; Kuchiki, D.; Caira, M.R. Optical resolution of medium-size lactones by inclusion crystallization with optically active host compounds: Remarkable odd–even effects on the chiral recognition. *Tetrahedron: Asymmetry* **2006**, *17*, 1678–1683.
40. Tanaka K.; Toda, F.; Mochizuki, E.; Yasui, N.; Kai, Y.; Miyahara, I.; Hirotsu, K. Enantioselective Single-Crystal-to-Single-Crystal Photodimerization of Coumarin and Thiocoumarin in Inclusion Complexes with Chiral Host Compounds. *Angew. Chem. Int. Ed.* **1999**, *38*, 3523–3525.
41. Nassimbeni, L.R.; Niven, M.L.; Tanaka, K.; Toda, F. On the optical resolution of bicyclic enones through host-guest complex formation: The crystallographic result. *J. Crystallogr. Spectrosc. Research* **1991**, *21*, 451-457.
42. Toda, F.; Tanaka, K.; Marks, D.; Goldberg, I. Optical resolution of bicyclo[2.2.1]heptanone, bicyclo[2.2.2]octanone, and bicyclo[3.2.1]octanone derivatives by inclusion complexation with optically active host compounds. *J. Org. Chem.* **1991**, *56*, 7332–7335.
43. Takemoto, Y.; Kuraoka, S.; Hamaue, N.; Aoe, K.; Hiramatsu, H.; Iwata, C. Enantioselective Cu-catalyzed 1,4-addition of Me_3Al to a 4,4-disubstituted cyclohexa-2,5-dienone. *Tetrahedron* **1996**, *52*, 14177–14188.
44. Lavy, T.; Sheynin, Y.; Kaftory, M. The effects of space formed by host molecules in inclusion compounds on the homogeneity/heterogeneity of the photoreaction in the solid state. *Eur. J. Org. Chem.* **2004**, *23*, 4802–4808.
45. Urbańczyk-Lipkowska, Z.; Yoshizawa, K.; Toyota, S.; Toda, F. Easy isolation of the enol form of acetylacetone as an inclusion complex with host compounds. *CrystEngComm* **2003**, *5*, 114–116.
46. Ghazali, N.F.; Ferreira, F.C.; White, A.J.P.; Livingston, A.G. Enantiomer separation by enantioselective inclusion complexation–organic solvent nanofiltration. *Tetrahedron Asymmetry*, **2006**, *17*, 1846–1852.
47. Olszewska, T.; Pyszno, A.; Milewska, M.J.; Gdaniec, M.; Polonski, T. Thioamides and selenoamides with chirality solely due to hindered rotation about the C–N bond: Enantioselective complexation with optically active hosts. *Tetrahedron Asymmetry*, **2005**, *16*, 3711–3717.
48. Olszewska, T.; Milewska, M.J.; Gdaniec, M.; Małuszyńska, H.; Połoński, T. Asymmetric Transformation of N-Nitrosamines by Inclusion Crystallization with Optically Active Hosts. *J. Org. Chem.* **2001**, *66*, 501–506.

49. Olszewska, T.; Sikorski, A.; Herman, A.; Połoński, T. Helicity discrimination in N,N′-dibenzoyl-1,2,3,4,7,8,9,10-octahydro- 1,10-phenanthrolines and their thiono- and selenocarbonyl analogues by inclusion complexation with chiral diols. *Org. Biomol. Chem.* **2013**, *11*, 7522–7529.
50. Toda, F.; Tanaka, K.; Foces-Foces, C.; Infantes, L.; Claramunt, R.M.; Lopez, C.; Elguero, J. Host-guest chemistry. The structure and proton disorder of the three-component crystal formed by 3(5)-methyl-4-nitropyrazole, (R, R)-(−)-trans-4,5-bis (hydroxydiphenylmethyl)-2,2-dimethyl-1,3-dioxolane and toluene. *J. Phys. Org. Chem.* **1996**, *9*, 611–618.
51. Hu, X.; Shan, Z.; Li, W. Second example for the heterocomplexation of chiral diols and complete disproportionation of enantiomers for non-racemic 2,3-O-cyclohexylidene-1,1,4,4-tetraphenylthreitols. *J. Fluor. Chem.* **2010**, *131*, 505–509.
52. Zhu, J.; Zhou, Z.-Y.; Fu, F.-M.; Deng, J.-G.; Mi, A.-Q.; Jiang, Y.-Z.; Chau, T.-Y. Crystal structure analysis of (R)-ethyl-2-pyridine sulfoxide and (4R,5R)-TADDOL clathrate [C7H9NOS·C31H30O4]. In *Gaodeng Xuexiao Huaxue Xuebao* 1999; Volume 20, p. 1081.
53. Bourne, S.A.; Oom, B.M.; Toda, F. Kinetics of desolvation from crystalline inclusion compounds of a diol host with methanol and ethanol. *J. Chem. Soc. Perkin Trans. 2* **1997**, *3*, 585–588.
54. Goldberg, I.; Stein, Z.; Weber, E.; Dorpinghaus, N.; Franken, S. Exploring the inclusion properties of new clathrate hosts derived from tartaric acid. X-Ray structural characterization of the free ligands and their selective interaction modes with alkylamine guests. *J. Chem. Soc. Perkin Trans. 2* **1990**, *16*, 953–963.
55. Barton, B.; Hosten, E.C.; Jooste, D.V. Comparative investigation of the inclusion preferences of optically pure versus racemic TADDOL hosts for pyridine and isomeric methylpyridine guests. *Tetrahedron* **2017**, *73*, 2662–2673, doi:10.1016/j.tet.2017.03.049.
56. Beck, A.K.; Bastani, B.; Plattner, D.A.; Petter, W.; Seebach, D.; Braunschweiger, H.; Gysi, P.; La Vecchia, L. Grossansätze zur Herstellung von $\alpha, \alpha, \alpha', \alpha'$-tetraaryl-1,3-dioxolan-4,5-dimethanolen(TADDOLe): Nützliche hilfsstoffe für die EPC-Synthese und ihre struktur im Festkörper. *Chimia* **1991**, *45*, 238–244.
